# Book Review of "Leprosy in Premodern Medicine. A Malady of the Whole Body" by Luke Demaitre PhD

**DOI:** 10.1186/1747-5341-2-24

**Published:** 2007-10-31

**Authors:** Karin M Schmitt

**Affiliations:** 1 Novartis Foundation for Sustainable Development, P.O. Box CH – 4002, Basel, Switzerland

## Abstract

Luke Demaitre's *Leprosy in Premodern Medicine: A Malady of the Whole Body *is a highly interesting study of the medical history of leprosy and the medical and social perceptions on leprosy that have been around for centuries. Remarkably, it is likely that leprosy will disappear from the face of the Earth in our generation, thanks to the development of a curative treatment and its increasing availability (although the battle has not yet been won completely). Demaitre's book is a very good read not only for its information about leprosy but also for all interested in or affected by the social phenomenon of stigma. In illnesses such as leprosy, HIV/AIDS, epilepsy, and mental disorders such as schizophrenia, the stigma attached to the condition may be worse than the condition itself.

## 

If we read Leviticus Chapter 13, it seems that leprosy once was such a dire affliction that even the LORD seemed to have no mercy on those who suffered from this disease. Here are the words of Leviticus in Chapter 13: "And the LORD spoke unto Moses and Aaron, saying, When a man shall have in the skin of his flesh a rising, a scab, or a bright spot, and it be in the skin of his flesh *like *the plague of leprosy; then he shall be brought unto Aaron the priest, or unto one of his sons the priests: And the priest shall look on the plague in the skin of the flesh: and *when *the hair in the plague is turned white, and the plague in sight *be *deeper than the skin of his flesh, it *is *a plague of leprosy: and the priest shall look on him, and pronounce him unclean."

Priests played for a long time a leading role in examination and diagnosis; it was only in the second half of the thirteenth century that learned physicians moved to the center stage of leprosy management. However, as leprosy was a "special" disease, icons of pre-modern medicine dealt with it much earlier and as profoundly as it was possible with the knowledge available at their time: Outstanding examples are Galen of Pergamum (ca. 129–200 AC), Avicenna (who, at around 1000 AC, under his Arab name Ibn Sina compiled the Arab Canon of Medicine which in its Latin translation by Gerard de Dremona became the standard medical encyclopedia of Europe's medieval time); as well as Bernard de Gordon (undisputed authority at the University of Montpellier in the 13th century).

The notion that leprosy is a *contagious and hereditary *disease – both characteristics that constitute a personal disaster for those afflicted – developed as a result of long-standing perceptions which stood the test of time well into the 18th century (p. 132 ff). The standard verdict was correct: Leprosy (then) was not curable – but this had dire consequences: The fear of transmission led at least to isolation, sometimes to torture and burning (as in the Kingdom of Aragon around 1320, where leprosy sufferers were accused of conspiring with Jews and Muslims to poison wells and rivers).

Over the centuries, a wide variety of issues were brought up as causes of leprosy (pp. 162 ff) – they ranged from "imbalance of the liver", "surfeit", "bad food" and "blocked pores" to "emotions" and "heredity". Not surprisingly, given the role of women in society in those days, "conception in menstruation" was seen as a contributing cause. The curative approaches and the medical prognosis reflected the understanding of the causes. Physical examination became increasingly a regular part of the diagnosis process only in the 13th century; checklists were developed to enable learned practitioners to do a proper examination. Looking at the list of signs "common to all forms of leprosy", according to Guy de Chauliac (1363), we find a number of characteristics that have much more to do with poverty, its consequences and the reaction of the social environment than with a particular disease, forget abut leprosy: "scabies", "fetor of breath", "terrible stare" or "nightmares".

For centuries, treatment recommendations were to keep patients from "salty, acidic, and spicy food, and from extremes of cold and heat"; other recommendations were "to keep away entirely from coitus" (p. 258). "Medicines" included "vinegar mixed with honey or sugar", but also "bloodletting and cathartics" as well as "seawater". For those who could afford a more expensive treatment – "paste-like sugary compounds, based on amber, pearls, and the like" (p. 262) were recommended. Of course – as is the case with all conclusions human beings draw from facts – personal mindsets and obsessions also left their trace in rather weird medical recommendations, be they to dry "the skin that is cut the circumcision of a child" and "mix it with a potion of a little musk" and eat it or administer "blood of toddlers" possibly mixed with "menstrual blood". (p. 268 f.)

Luke Demaitre wrote a highly interesting and fascinating book. He looked into the development of the understanding of a disease that was (and in some places of this Earth still is) frightening people. He gives account of the many societal perceptions of and human attitudes towards an ancient scourge that has, at some stage, afflicted every country in the world. His book reveals that leprosy was never "just a disease". It was a "malady of the whole body" as well as a "disease of the soul" – with disastrous consequences for those who became infected. Of course, there were different formal definitions, regional differences, a diversity of social settings and a variety of medical concepts, and hence there were ambiguities and other aspects of undesirable human behavior such as, in doubt, giving in to social pressures and err on the side of diagnosing a patient to be a leper rather than not. This in a time of mass poverty, in which people who could not afford a high level of personal hygiene ended up with eczemas and scabies rather than leprosy.

But there is also a very consolatory fact: Once the management of leprosy was given into the hands of professional medical practitioners, their work resulted in moderating society's fears and rejection. In a situation where cure was not possible, medical practitioners did their palliative best to prolong life, ease suffering, and strive to preserve some residua of well being in their patients.

In premodern times – and as a matter of fact in some parts of the world still today – leprosy was more than just an infectious disease. It was considered a shameful affliction, brought about by wrongdoing in a previous life, through a curse of God or witchcraft; in other words, a punishment of all sorts. Sufferers were ashamed of their condition and tried to hide the disease for fear of social repercussions. Yet lack of treatment, or even delayed treatment, merely served to increase the risk of disabilities which, in turn, strengthened and perpetuated the stigma of the disease – a vicious circle. Only in 1873, G. Armauer Hansen identified the causative organism (mycobacterium leprae) of the disease – a tough enemy of human happiness that can survive for several months in the soil and, slowly multiplying, affect the peripheral nerves, the skin, and eventually the bones. For reasons hitherto unknown, less than 5 percent of most populations are susceptible, and of those infected the individual immune response varies widely.

The author disproves a lot of wrong "common knowledge" and "diehard simplifications" about the response to the disease in Europe, the role of the Crusades in the spread of the diseases, and other prejudices. Never before has so much work been invested in the history of leprosy – and this is very much appreciated at a time where it approaches global elimination.

Only in the early 1980s, the face of leprosy could change dramatically thanks to the development of a curative treatment and its increasing availability, free of charge, to patients. Multi-Drug Therapy (MDT), which is the treatment recommended by the World Health Organization (WHO), cures patients, interrupts the transmission of leprosy, and prevents disabilities. Even patients with the severest form of the disease experience visible clinical improvement within weeks of starting treatment. The drugs are given for free by the pharmaceutical company Novartis.

It remains to be hoped that a second book is written to tell the fascinating public health success story of the fight against this biblical disease in a similar excellent way: Leprosy is on the verge of being eliminated, because scientists, politicians, professionals from the World Health Organization as well as from NGOs and private-sector foundations, and a pharmaceutical company did what was the right thing to do: Bundle their efforts for a final push. Making leprosy diagnosis and free treatment available at the village level within primary health facilities in areas where the disease occurs has proved to be a highly effective way of bringing treatment closer to patients. As communities witness the impact of innovative treatment, age-old prejudices have begun to change and, with them, societal norms. Discriminating customs are fading in communities that have seen people cured through the multi-drug-therapy developed by Novartis and recommended by the World Health Organization. Around the world, efforts are being made to change the image of leprosy and encourage people to seek timely treatment. To remove the stigma associated with deformities, their prevention, correction and eventual rehabilitation are being integrated into general health services. As a result, hopelessness and despair are giving way to the idea that leprosy can be just another chapter in a person's life.

Today, tremendous progress has been made, but the battle has not yet been won completely. Tackling residual problems requires learning from past successes and failures, as well as having a clear understanding of the remaining obstacles. In January 2008, the final International Leprosy Congress will be held in Hyderabad – in our generation, leprosy will disappear from the face of the Earth – a disease that once was such a dire affliction that beggars found great profit in counterfeiting it so they could raise more money.

Luke Demaitre's book is an outstanding study of the medical history of leprosy and the medical and social perceptions on leprosy that have been around for centuries. The author is a medical historian, a visiting professor of history in the Humanities in Medicine Program at the University of Virginia. This book is a very good read not only for representatives of his own discipline but for all interested in or affected by the social phenomenon of stigma. In illnesses such as leprosy, HIV/AIDS, epilepsy, and mental disorders such as schizophrenia, the stigma attached to the condition may be worse than the condition itself.

## Competing interests

The author is employed by the Novartis Foundation for Sustainable Development in Basel, Switzerland.

